# Enhancing percutaneous pedicle screw fixation with hydroxyapatite granules: A biomechanical study using an osteoporotic bone model

**DOI:** 10.1371/journal.pone.0223106

**Published:** 2019-09-26

**Authors:** Haruo Kanno, Toshimi Aizawa, Ko Hashimoto, Eiji Itoi

**Affiliations:** Department of Orthopedic Surgery, Tohoku University School of Medicine, Sendai, Japan; University of California Davis, UNITED STATES

## Abstract

**Introduction:**

Percutaneous pedicle screw (PPS) can provide internal fixation of the thoracolumbar spine through a minimally invasive surgical procedure. PPS fixation has been widely used to treat various spinal diseases. Rigid fixation of PPS is essential for managing osteoporotic spine in order to prevent the risks of screw loosening and implant failure. We recently developed a novel augmentation method using hydroxyapatite (HA) granules for PPS fixation. The aim of this study was to evaluate the strength and stiffness of PPS fixation augmented with HA granules using an osteoporotic bone model.

**Methods:**

Screws were inserted into uniform synthetic bone (sawbones) with and without augmentation. The uniaxial pullout strength and insertion torque of the screws were evaluated. In addition, each screw underwent cyclic toggling under incrementally increasing physiological loads until 2 mm of screwhead displacement occurred. The maximal pullout strength (N), maximal insertion torque (N·cm), number of toggle cycles and maximal load (N) required to achieve 2-mm screwhead displacement were compared between the screws with and without augmentation.

**Results:**

The maximal pullout strength was significantly stronger for screws with augmentation than for those without augmentation (302 ± 19 N vs. 254 ± 17 N, p < 0.05). In addition, the maximal insertion torque was significantly increased in screws with augmentation compared to those without augmentation (48 ± 4 N·cm vs. 26 ± 5 N·cm, p < 0.05). Furthermore, the number of toggle cycles and the maximal load required to reach 2 mm of displacement were significantly greater in screws with augmentation than in those without augmentation (106 ± 9 vs. 52 ± 10 cycles; 152 ± 4 N vs. 124 ± 5 N, p < 0.05).

**Conclusions:**

Augmentation using HA granules significantly enhanced the rigidity of PPS fixation in the osteoporotic bone model. The present study suggested that novel augmentation with HA granules may be a useful technique for PPS fixation in patients with osteoporotic spine.

## Introduction

Osteoporotic spine is becoming increasingly common as the population ages.[1, 2] Spinal surgeries with pedicle screw fixation for osteoporotic spine have risks of screw loosening and back-out, which may lead to a loss of correction, nonunion and implant failure.[3] It has been reported that the rate of screw loosening ranges from <0.6% to 15% in non-osteoporotic patients and even up to 60% in osteoporotic subjects.[4, 5] Previous studies have shown that a decrease in the bone mineral density (BMD) in the spine significantly increases the incidence of screw loosening and non-union after spinal fusion surgeries.[6, 7] Therefore, it is important to consider effective ways to augment screw fixation in patients with osteoporosis in order to achieve optimal postoperative clinical outcomes. In conventional pedicle screw fixation, various augmentation techniques have been widely used for osteoporotic spine, including additional hook,[8–10] sublaminar band/tape,[11, 12] cement augmentation[13, 14] and expandable[15, 16] or hydroxyapatite (HA)-coated screws.[17, 18] Many studies have demonstrated the biomechanical advantages of these augmentation methods.[19, 20]

Previous studies indicated that placement of substances into the tapped screw hole increases the bone-metal interface friction force and enhances mechanical strength of screw fixation.[21–27] Osteoconductive ceramic bone graft substitutes, such as HA and calcium phosphate (CaP), have received attention as clinically applicable biomaterials to improve the stability of screw fixation.[21, 27] The structures of these biomaterials consist of interconnective micropores as cancellous bone and enable mesenchymal cell migration and differentiation into osteoblasts within the micropores.[28] The HA graft material can be slowly degraded *in vivo* after implantation and consequently induce new bone formation and remodeling for optimal mechanical strength without interference.[23, 27] The HA graft material having more crystallization and higher density provides greater mechanical strength and resistance to degradation, and leads to the long-term stability.[21, 27]

Recently, minimally invasive techniques have become popular in spinal surgery.[29, 30] Percutaneous pedicle screws (PPS) can provide internal fixation with a minimally invasive technique and are now widely used to treat various spinal pathologies, including trauma, tumors, infection, deformity and degenerative disease.[30, 31] The less-invasive procedure of PPS fixation offers less damage to the surrounding tissues and a decrease in intraoperative blood loss, postoperative pain and recovery time.[32–34] However, PPS fixation has potential disadvantages with respect to obtaining rigid stabilization and bone union compared with conventional pedicle screw fixation. Bone grafting to achieve posterior or posterolateral bone fusion of the spine is generally impossible in PPS fixation because a small incision is used for the percutaneous procedure.[29, 35] Furthermore, transverse connectors between the rods cannot be added in order to enhance the rigidity of PPS fixation.[36, 37] In fact, PPS fixation carries a certain risk of screw loosening and implant failure, with the rate of screw loosening ranging from 11% to 20%.[38–40] However, no well-established augmentation method is available at present for PPS fixation.

To overcome these disadvantages of PPS fixation, we recently developed a novel augmentation method using HA granules.[41] We also created an original insertor of the HA granules to achieve this augmentation percutaneously without any additional skin incision or tissue damage. This method can be applied to PPS fixation for various spinal surgeries in patients with osteoporosis.[41] However, the mechanical performance of this method for enhancing PPS fixation has not been investigated. The purpose of the present study was to perform biomechanical analyses to evaluate the strength and stiffness of PPS fixation augmented with HA granules using an osteoporotic bone model.

## Materials and methods

Independent Ethics Committee of Tohoku University School of Medicine approved this study. The written consent was obtained from all donors.

### Synthetic bone model

Various types of rigid polyurethane foams have been used as a substitute material for cadaveric bones because they have homogenous structure and consistent material properties, and are easily available.[42, 43] This study used the standardized rigid polyurethane foam (Sawbones; 1522–23: pcf 5; Pacific Research Laboratories, Vashon Island, WA, USA). In order to simulate severe osteoporotic bone in mechanical testing, we used the polyurethane foam having a density of 0.08 g/cm^3^.[44, 45] To our knowledge, this density is the lowest among the solid rigid polyurethane foams that are commercially available and enable to simulate the severe osteoporotic cancellous bone.[46] The representative densities and type of the polyurethane foam were standardized and regulated by the American Society of Testing Materials (ASTM) Protocol.[47] According to the ASTM protocol, the uniformity and consistent properties of rigid polyurethane foam make it an ideal material for comparative testing of bones screws and other medical devices and instruments.[47] In many previous studies, polyurethane foam has been used for simulating the response of cancellous bone in various biomechanical tests of screws and provided consistent experimental results.[42, 43, 48–50] To obtain more consistent and reliable results of mechanical testing, the polyurethane foam was used in this study. The polyurethane foam was cut to a size of 43 mm in length, 60 mm in width and 40 mm in height for mechanical testing.

### Screw placement and augmentation

**Fig 1:**
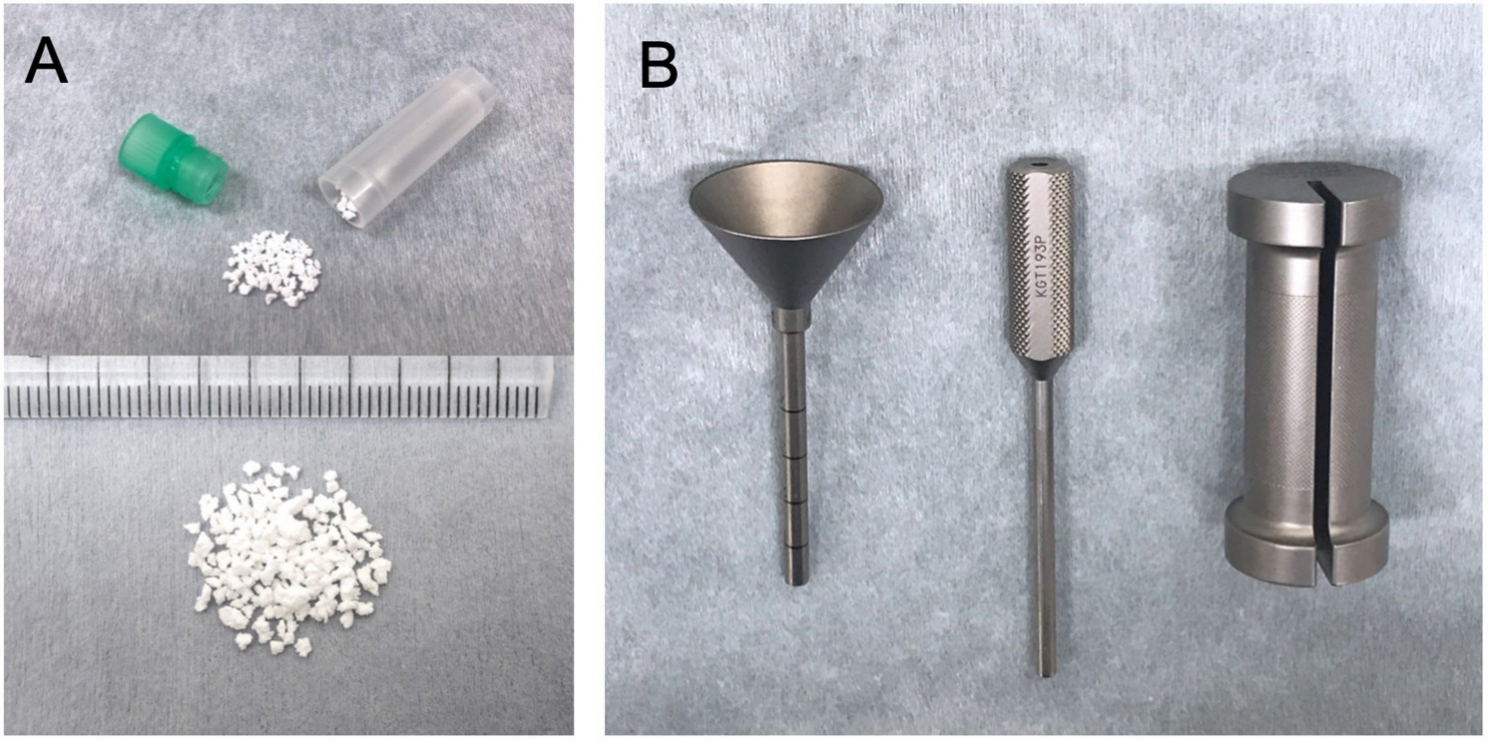
HA granules and the insertion device for the augmentation of PPS fixation. Commercially available HA granules were used for augmentation (A). The HA insertion device consisted of a funnel-shaped external cylinder, internal cylinder and slide hammer (B). The tip of the external cylinder is 5.5 mm in diameter.

**Fig 2:**
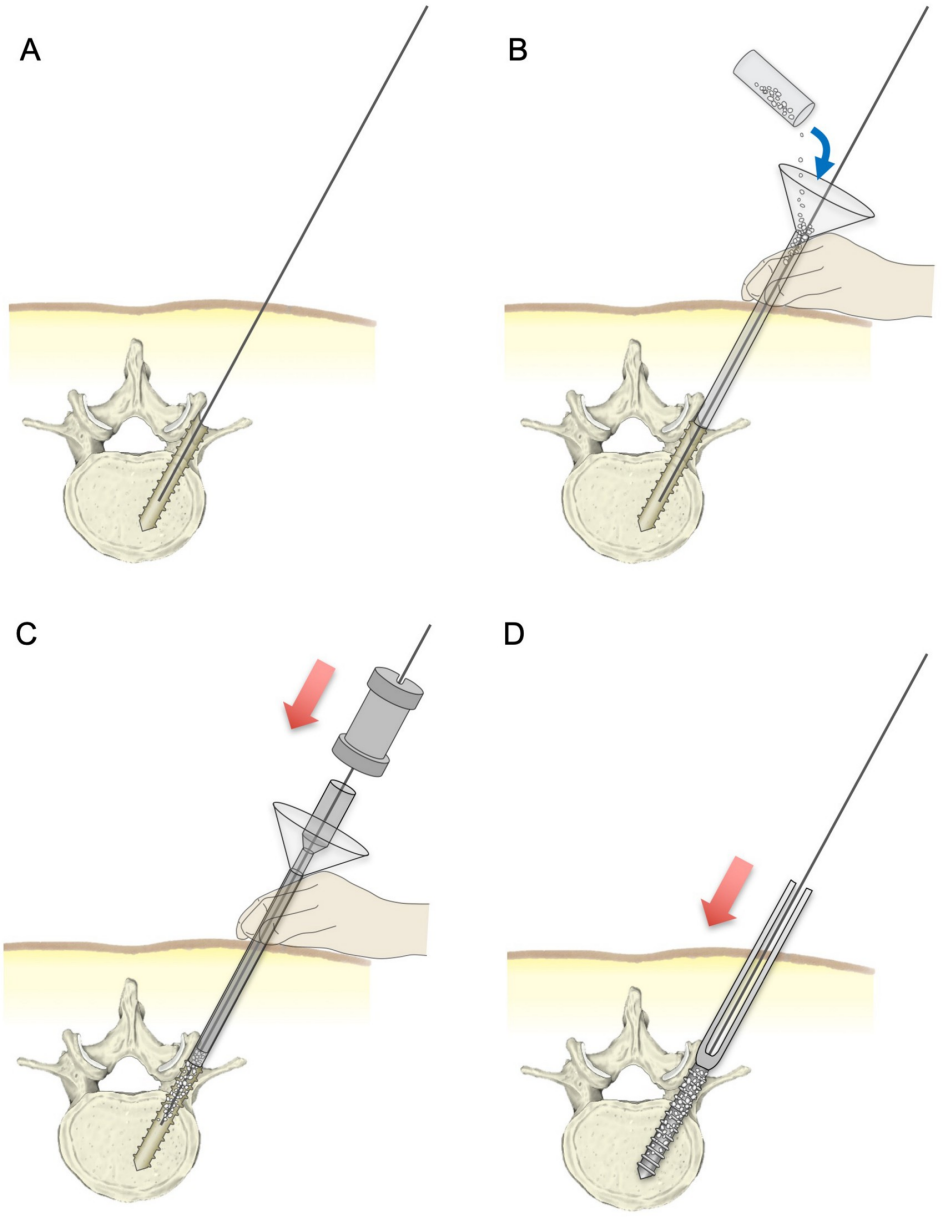
Surgical procedures of augmentation of PPS fixation with HA granules. A guidewire was inserted into the vertebra, and tapping was performed as a standard procedure of PPS insertion (A). The funnel-shaped external cylinder was placed at the screw hole along the guidewire. HA granules were inserted into the external cylinder (B). The granules were then pushed into the screw hole using the internal cylinder and slide hammer (C). Finally, the cannulated PPS was placed into the screw hole (D).

To perform the augmentation of PPS, we developed a dedicated device to insert HA granules percutaneously into the screw hole (Fig 1).[41] Commercially available HA granules (50% porosity, 1–2 mm in particle size; APACERAM, PENTAX Corp., Tokyo, Japan)[51] were used for the augmentation. In this study, 0.25 g of granules, which is equivalent to the amount of HA within an HA stick (PENTAX Corp.),[25, 41] was used for the augmentation of each screwing. The actual surgical procedures of this augmentation are briefly described in Fig 2.

In the present study, the prepared polyurethane foam was securely fixed to a testing apparatus using a metal clamp, and then screw placement with augmentation was performed. According to the standard procedure of PPS fixation,[35, 52] the Jamshidi needle was inserted 20 mm into the foam. The shaft of the needle should be perpendicular to the surface of the foam. Once a guidewire was inserted into the foam through the needle, the tapping procedure was performed with a 6.5-mm tap. In order to introduce the HA granules into the tapped screw hole, the funnel-shaped external cylinder of the insertion device was placed at the entry point of the screw hole along the guidewire. The HA granules were inserted in the funnel and then pushed into the screw hole using the internal cylinder and slide hammer. Finally, the cannulated PPS (6.5 mm in diameter, 40 mm in length; Voyager; Medtronic Sofamor Danek, Co., Ltd., Osaka, Japan) was placed into the screw hole until the polyaxial screwhead made contact with the foam surface.

### Mechanical testing

Fifty prepared synthetic bones were used for screw pullout testing (n = 20), the measurement of the screw insertion torque (n = 20) and screw toggle testing (n = 10). All screw placements and augmentation procedures were performed by the same surgeon.

**Fig 3:**
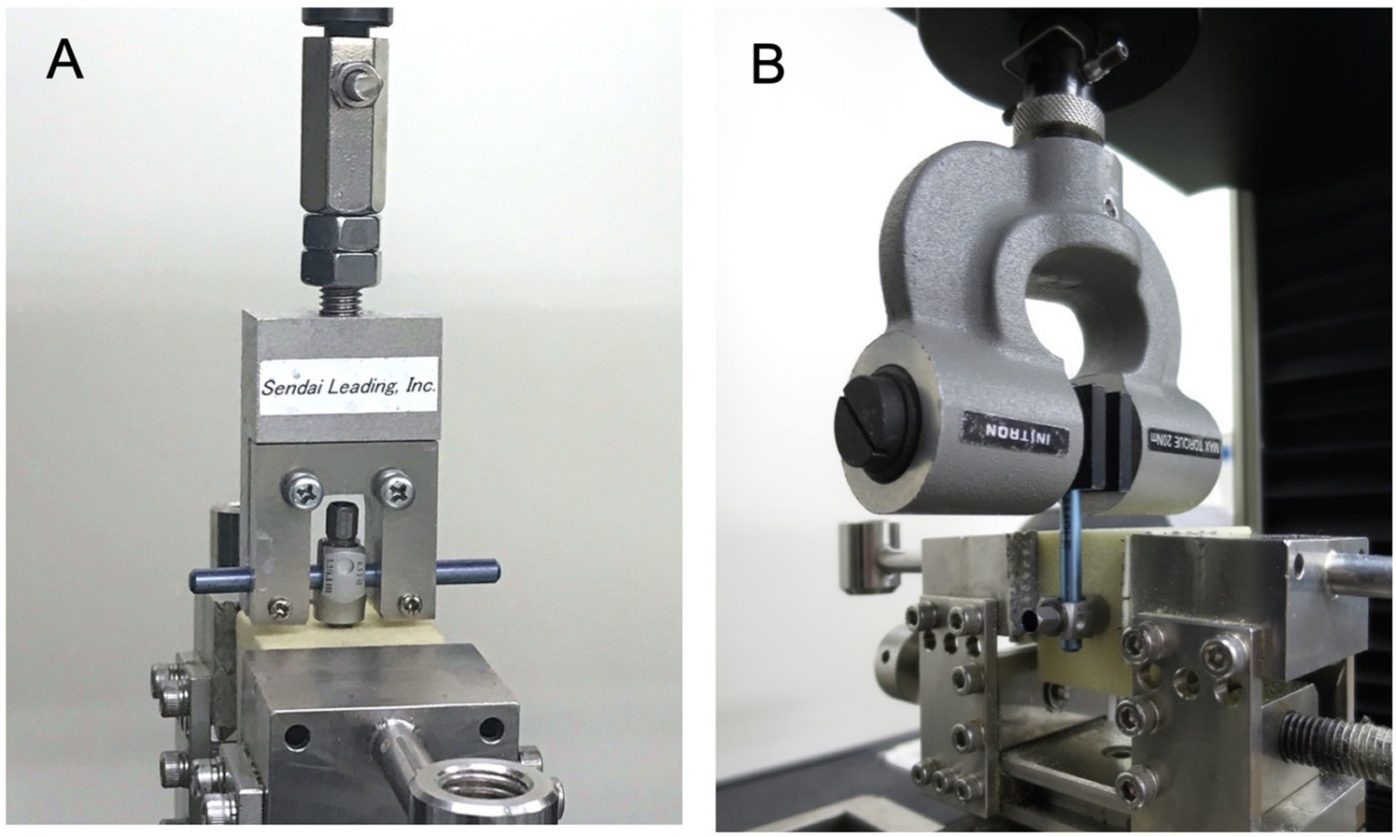
Biomechanical study setup for uniaxial pullout testing (A) and toggle testing (B).

The pullout testing for the screws with and without augmentation was performed as described previously.[15, 43, 53, 54] The pullout strength of the screws was evaluated using a universal mechanical testing machine (5566; Instron Japan Co., Ltd., Kawasaki, Japan) (Fig 3A). The screwhead was connected to a 5.5-mm diameter rod using a setscrew. The foam was securely fixed to the base of the testing machine and aligned so that the rod was parallel to the base. A custom-made jig connected to the testing machine was then attached to the rod so that the direction of the screw was collinear to the pullout force. All screws were withdrawn uniaxially at a rate of 5 mm/min until the screw was completely out.[15] In each testing session, the force-displacement data were recorded continuously at a sample rate of 50 Hz using a computerized data collection system (Bluehill 3^®^; Instron Japan Co., Ltd.). The force-displacement curve was analyzed in order to determine the maximal pullout strength and stiffness of screw fixation. The maximal pullout force was calculated as the largest load experienced during each test. The stiffness was defined as the slope of the most linear part of the load-displacement curve before the yield point.[55]

During the screw insertion, the maximal insertion torque was measured using a calibrated torque wrench with a range of 5 to 200 N·cm (Kanon DPSK-N20; Nakamura, Tokyo, Japan) and a custom adapter for the screwdriver and torque wrench.[6, 56] The peak value of the insertion torque for each screw was recorded.

To perform toggle testing, each screwhead was connected to a 5.5-mm diameter rod, and then the foam was securely fixed to a custom testing apparatus (Fig 3B). The connecting rod was oriented perpendicular to the screw axis and fixed to a special grip attached to the load cell. During the toggle testing, sagittally cyclic loads were applied to the screw using the mechanical testing machine (5566; Instron Japan Co., Ltd.).[57, 58] According to previous studies,[26, 57, 59] the force applied started at ± 100 N and increased by 25 N every 20 cycles to simulate the physiological condition of a pedicle screw placed in the lumbar spine. Cyclic toggling of the screws terminated once a total sagittal crosshead displacement of 2 mm was detected.[57, 60] The number of toggle cycles and the maximal load required to achieve 2-mm displacement were compared between screws with and without augmentation.

### Cadaveric study

To estimate clinical significance of the screw augmentation using HA granules, we further performed the screw pullout test using human cadaveric lumbar spines. Ten vertebrae (L1-L5) were collected from two fresh-frozen cadavers (ages: 72 and 89 years). The clinical history and images of whole-body computed tomography were reviewed to exclude the specimens having infectious, traumatic, neoplastic, congenic, developmental conditions or history of spinal surgery. Before mechanical testing, each lumbar vertebra was skeletonized and disarticulated. For mechanical testing, the vertebra was securely fixed to a testing apparatus, and then screw placement with augmentation was performed as described above. To minimize bias due to inter-individual and inter-vertebral variations in size, morphometry and bone density, the mechanical testing was performed in a paired array comparing each screw on the ipsilateral side with that on the contralateral side.[13, 61] As described above, the screw pullout test was performed to measure the maximal pullout force.

### Statistical analyses

The sample size estimation of this study was carried out based on the results of a similar experiment.[62] The sample size was calculated using a statistical power analysis software (G-Power version 3.1.9.4; Franz Faul, University Duesseldorf, Germany). The sample size calculation estimated that a minimum of 7 samples per group were required to obtain a power of 0.80 and an alpha value of 0.05 (two-sided). In the present study, the maximal pullout strength, stiffness and maximal insertion torque were statistically compared between screws with and without augmentation. The number of toggle cycles and the maximal force to achieve a 2-mm displacement of screwhead were also compared between the screws with and without augmentation. All data are shown as the mean ± standard deviation. Statistical significance was analyzed using the unpaired *t*-test. All statistical analyses were performed using the StatView Version 5.0 software program (SPSS, Chicago, IL, USA). Statistical results at p < 0.05 were considered significant.

## Results

**Fig 4:**
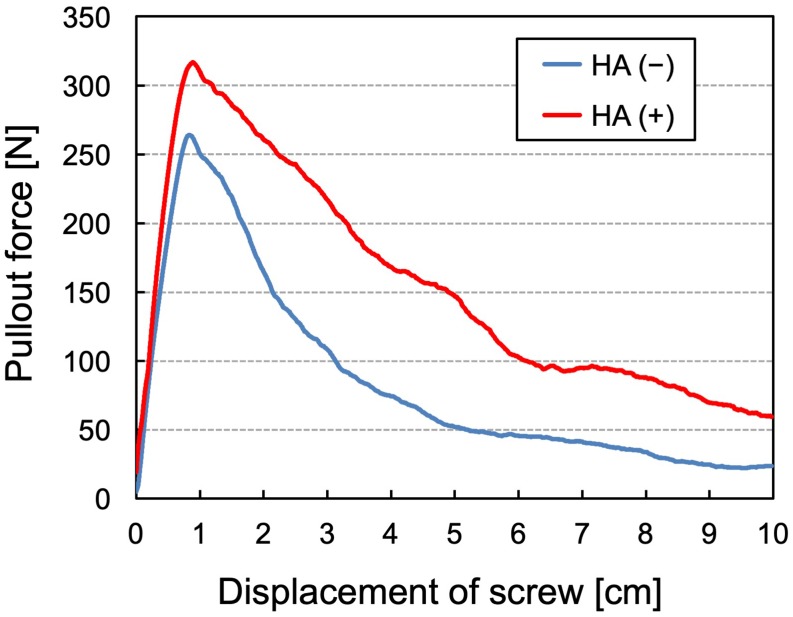
Representative load-displacement curves for uniaxial pullout tests of screws with and without augmentation. HA (+) and HA (−) indicate the screws with and without augmentation, respectively.

**Fig 5:**
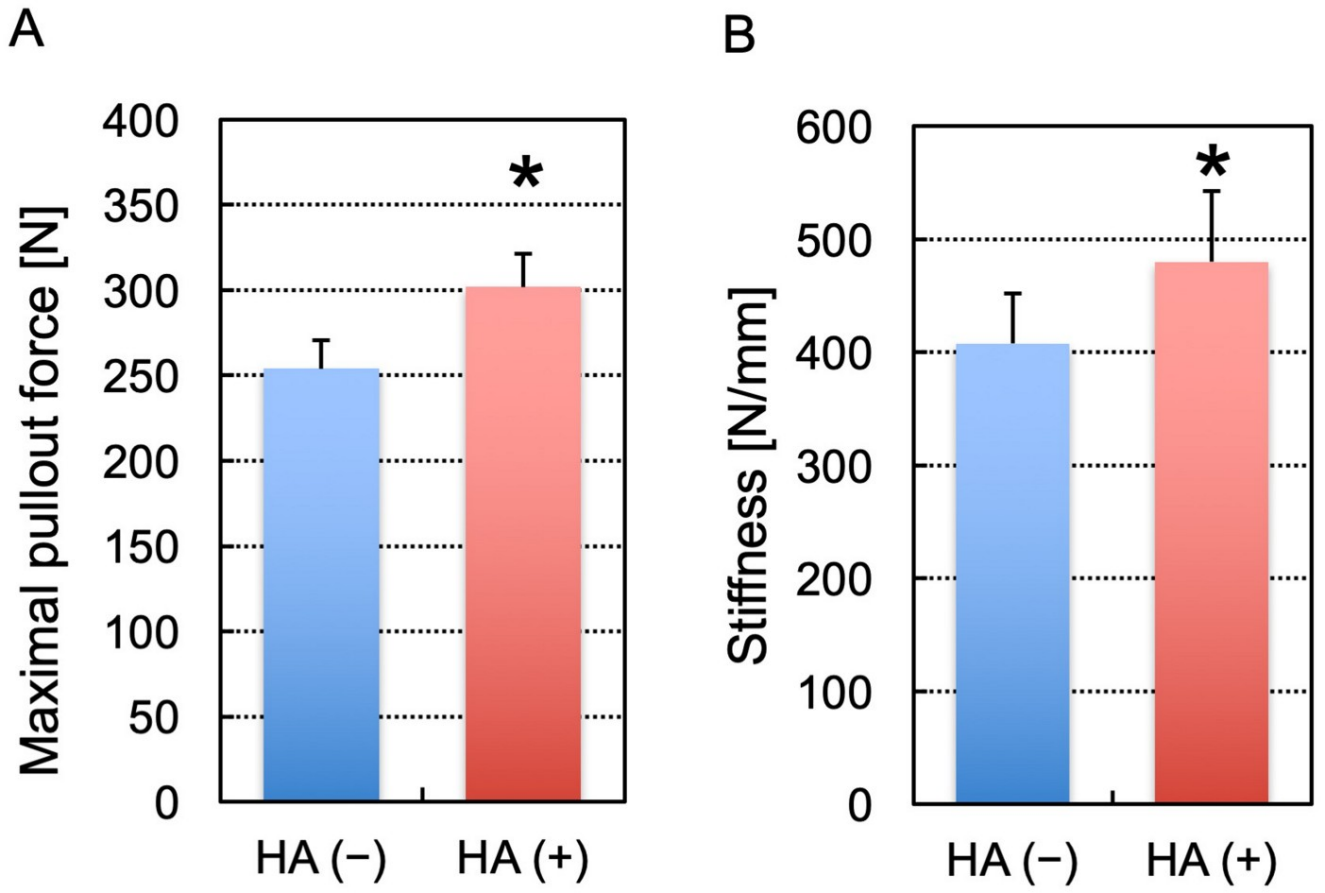
A comparison of the maximal pullout force (A) and stiffness (B) of screws. HA (+) and HA (−) indicate the screws with and without augmentation, respectively (n = 10 per group). The asterisk indicates a p value of < 0.05. The error bars indicate the standard deviation.

Representative load-displacement curves for the uniaxial pullout test of screws are shown in Fig 4. The maximal pullout strength was 302 ± 19 N for screws with augmentation and 254 ± 17 N for those without augmentation (Fig 5A). The maximal pullout strength was significantly greater in the screws with augmentation than in those without augmentation (*P* < 0.0001). In addition, the construct stiffness was 480 ± 63 N/mm for screws with augmentation and 408 ± 45 N/mm for those without augmentation (Fig 5B). A significant difference was noted in the stiffness between the screws with and without augmentation (*P* = 0.0082).

**Fig 6:**
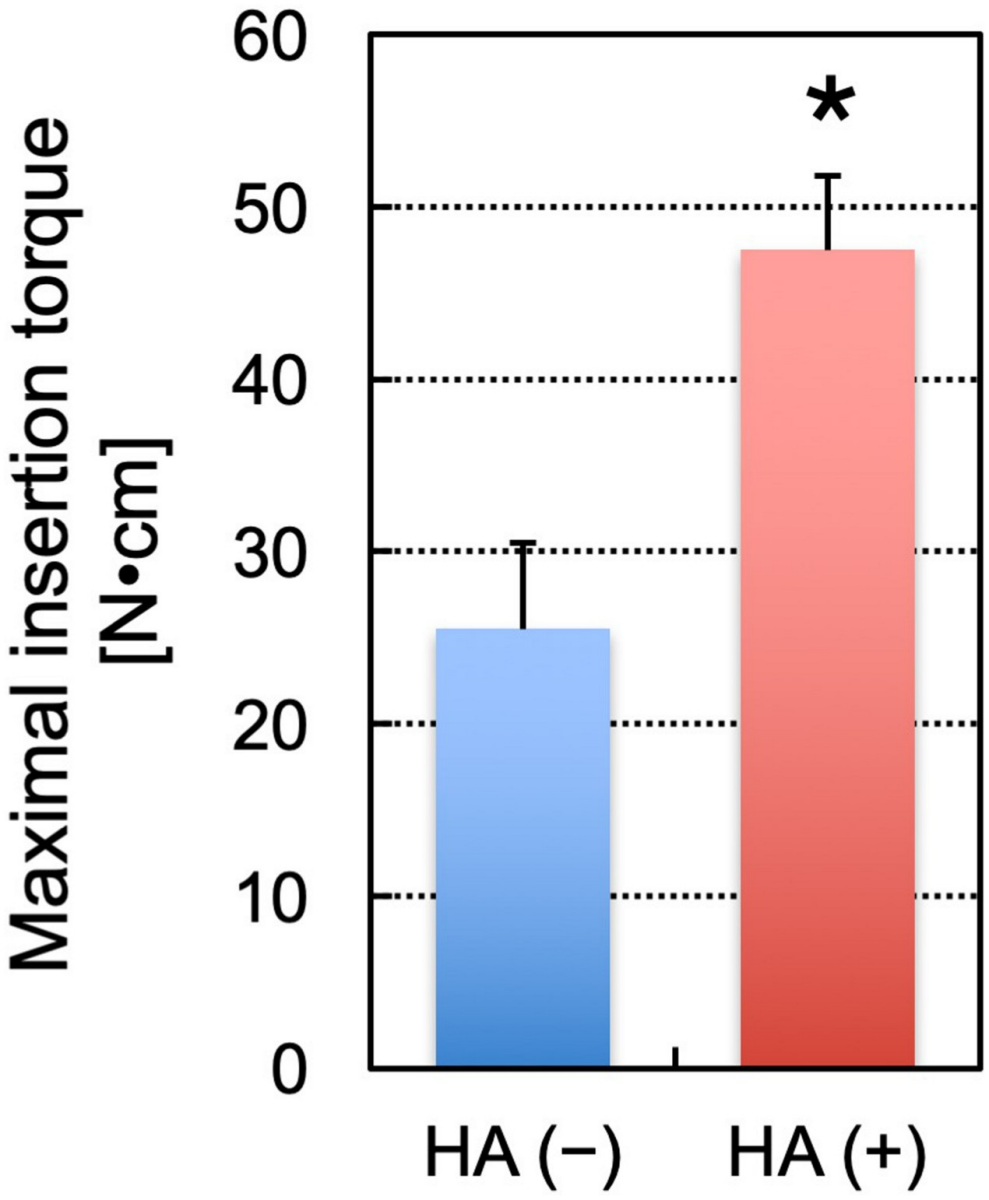
A comparison of the maximal insertion torque of screws. HA (+) and HA (−) indicate the screws with and without augmentation, respectively (n = 10 per group). The asterisk indicates a p value of < 0.05. The error bars indicate the standard deviation.

In the measurement of the screw insertion torque (Fig 6), the maximal insertion torque was significantly stronger in the screws with augmentation (47.5 ± 4.2 N·cm) than in those without augmentation (25.5 ± 5.0 N·cm) (*P* < 0.0001).

**Fig 7:**
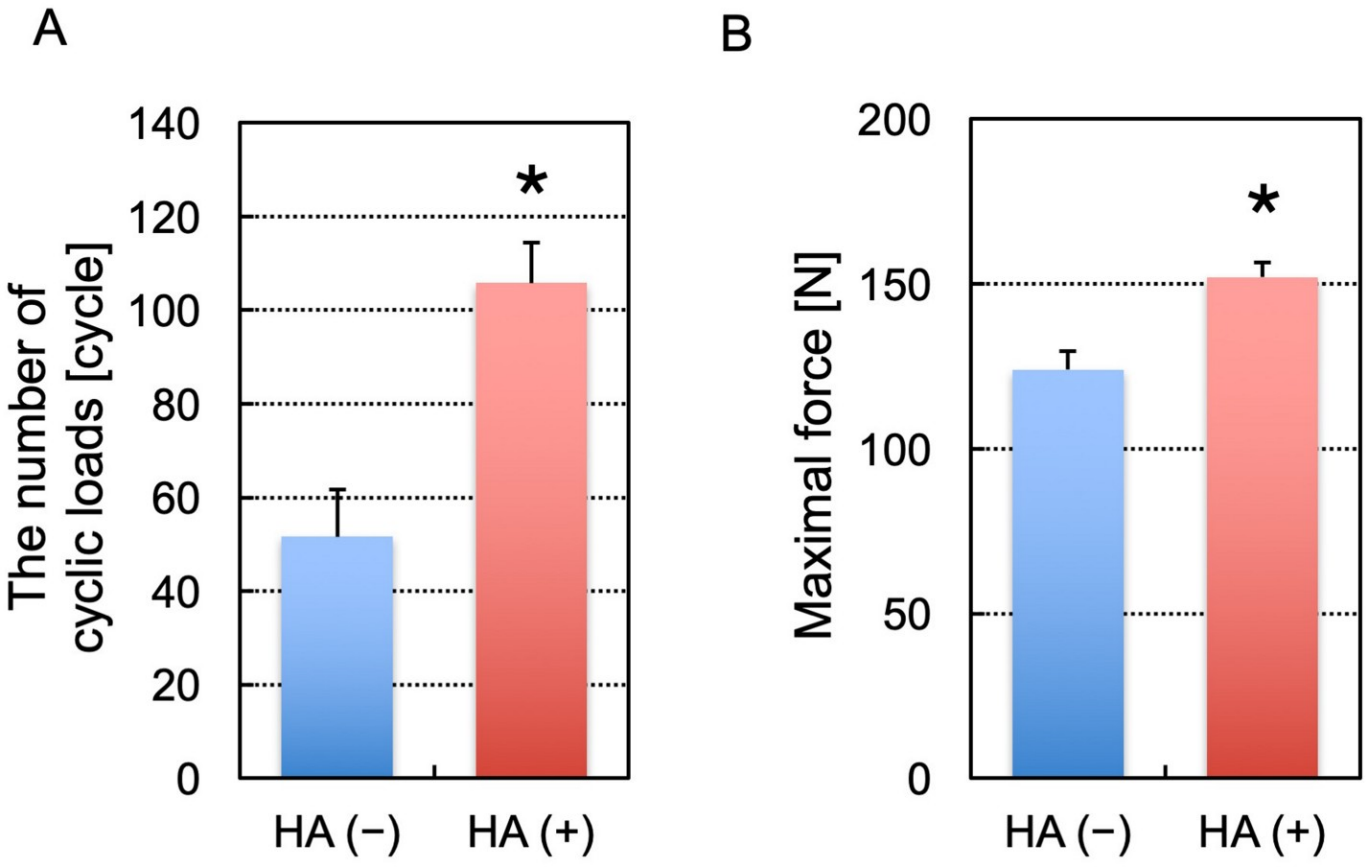
A comparison of the number of cyclic loads (A) and the maximal force (B) to reach the endpoint in toggle testing. HA (+) and HA (−) indicate the screws with and without augmentation, respectively (n = 5 per group). The asterisk indicates a p value of < 0.05. The error bars indicate the standard deviation.

The number of toggle cycles to reach 2 mm of screwhead displacement was 106 ± 8.7 cycles for screws with augmentation and 52 ± 10 cycles for those without augmentation (Fig 7). There was a significant difference in the number of toggle cycles required to achieve 2 mm displacement between the screws with and without augmentation (*P* < 0.0001). In addition, the maximal force required to reach this endpoint was also significantly greater in the screws with augmentation (152 ± 4.5 N) than in those without augmentation (124 ± 5.5 N) (*P* < 0.0001).

In the results of the cadaveric study, the maximal pullout strength was 282 ± 58 N for screws with augmentation and 192 ± 55 N for those without augmentation. The maximal pullout strength in the screws with augmentation was significantly greater than that in the screws without augmentation (*P* < 0.01).

## Discussion

To date, no useful augmentation method for PPS fixation that can be performed as a minimally invasive procedure has been proposed. The present study first demonstrated that augmentation with HA granules provided a significantly stronger pullout strength and stiffness of screw fixation than no augmentation in an osteoporotic bone model. The screw insertion torque was also significantly increased by the augmentation. In addition, the number of cyclic loadings to reach 2 mm of screwhead displacement was significantly greater in the screws with augmentation than in those without augmentation. The maximal force required to reach this endpoint was significantly increased in the screws with augmentation. Furthermore, the human cadaveric study using the lumbar vertebrae confirmed that the screw pullout strength was significantly enhanced by the augmentation. These results suggested that augmentation using HA granules may be an effective method of enhancing the rigidity of PPS fixation in the osteoporotic spine.

A number of previous studies have performed only uniaxially pullout tests to evaluate the mechanical performance of pedicle screw fixation.[26, 61, 63] However, the data of uniaxial pullout tests should be cautiously interpreted because implant failure after pedicle screw fixation rarely occurs in this manner *in vivo*. Pedicle screws placed in the lumbar spine are actually loaded with forces in various directions during flexion/extension, rotation and lateral bending of the patient’s body trunk. Another way to examine the pedicle screw stability in a more physiological manner is toggle testing, wherein loads in different directions are repetitively applied to the screw.[57–59] In addition, the maximal insertion torque of pedicle screw has been shown to be significantly correlated with the BMD of the spine and the mechanical stability against cyclic tilting moment.[56] Therefore, the present study aimed to clarify various mechanical properties of the augmentation of PPS with HA granules under physiological loads on the osteoporotic bone model. The current study analyzed not only the pullout strength but also the maximal insertion torque and the resistance to cyclic toggling of the screws. Our results showed that augmentation using HA granules significantly increased the maximal pullout strength, the maximal insertion torque and the resistance to cyclic toggling of the screws compared to those without augmentation. The present study therefore provided valuable data supporting the effectiveness of augmentation using HA granules for PPS fixation.

Osteoporosis is a major public health problem for aging populations, and its prevalence is predicted to increase further in various countries.[1, 64, 65] Aging is a significant risk factor of major medical complications in patients undergoing spinal surgeries.[66, 67] Previous studies have suggested that minimally invasive spinal surgeries using PPS fixation may reduce the incidence of complications in elderly patients.[39, 68, 69] Minimally invasive spinal surgeries with PPS may become an optimal treatment option for reducing complication rates.[30, 68] However, the prevalence of spinal osteoporosis and implant failure related to bone fragility increases with age.[2] Therefore, an effective and useful augmentation method of PPS fixation is required in order to prevent osteoporosis-related complications in elderly patients undergoing minimally invasive surgeries. The results of this study showed that the augmentation with HA granules significantly enhanced the mechanical strength of screw fixation in the osteoporotic bone model as well as cadaveric spine. The present study suggested that augmentation using HA granules can be a useful minimally invasive technique for PPS fixation in patients with osteoporosis. More rigid PPS fixation with effective augmentation may reduce the risk of screw loosening and implant failure and provide better postoperative clinical outcomes in elderly patients. Further studies in clinical settings will provide evidence that this augmentation approach actually reduces the incidence of screw loosening or implant failure following PPS fixation in patients with osteoporosis.

Spinal surgeries with instrumentation for osteoporotic spine are challenging due to the poor mechanical properties of osteoporotic bone.[3, 20, 70] In traditional open spinal surgeries, various augmentation methods have been applied to increase the stability of pedicle screw fixation. The use of supplemental hooks or sublaminar bands with pedicle screw enhances the stability of fixation.[8, 11, 12] Expandable pedicle screws increase the screw pullout strength compared with standard screws.[15, 16] Pedicle screw augmentation using cement such as PMMA improves the fixation strength of spinal instrumentation.[13, 14] Several previous studies have suggested that HA stick or HA grunt inserted into the tapped screw hole may help enhance the initial strength of pedicle screw fixation.[22, 25, 71] The placement of HA into the screw hole increases the bone-metal interface friction force and reinforces the screw stability.[21] In contrast, the application of PPS fixation significantly limits the utility of these conventional augmentation methods because of the small incision and narrow surgical field. Furthermore, the insertion of materials, such as PMMA, into the screw hole for augmentation is difficult in PPS fixation because a guidewire is placed within the screw hole and surgeons are unable to see the screw entry point in the operative field. Importantly, the original device used in the current study to insert HA granules into the screw hole enabled the percutaneous augmentation of PPS. Indeed, we have now performed this augmentation for PPS fixation in various minimally invasive spinal surgeries without any additional skin incision or tissue damage.[41] We recently reported that the incidence of screw loosening at one-year follow-up was only 6.8% (5/74 screws) in osteoporotic patients who underwent PPS fixation with the augmentation using HA granules.[41] On the other hand, Ohba et al. reported that incidence of PPS loosening without augmentation was 15.2% (44/290 screws) at one year after surgery.[38] In addition, Ohtori et al. showed that 25.5% (26/102 screws) of conventional pedicle screws implanted in patients with osteoporotic spine became loose at 12-month follow-up.[72] Thus, these findings suggested that the augmentation using HA granules might be clinically useful for reducing the incidence of screw loosening in patients having osteoporotic spine.

Augmentation using cement and expandable screws reportedly has several disadvantages, specifically with regard to the shape and nature of the cement and screws.[4] Cement-based augmentation carries a risk of cement extravasation, which can lead to neurological and cardiovascular complications.[73] Cements, including PMMA, have exothermic properties that may induce bone necrosis and degeneration of adjacent discs.[73, 74] In addition, PMMA has no potential for bone remodeling, osteoinduction, osteoconduction or osteointegration and may block the vascular supply by its presence. Furthermore, cement augmentation may increase the risk of fracture of the vertebra during screw removal.[75] Expandable pedicle screws also carry a similar risk of vertebral bone destruction during screw removal, which may cause nerve root or dural injury.[76] In contrast, the application of HA granules for screw augmentation can prevent these risks because HA has a high bioaffinity and biocompatibility. HA has been shown to promote osteoconduction and osteointegration and is slowly replaced by the host bone.[28] HA induces no exothermic reaction or toxic effects that might damage the surrounding tissue following implantation. Overall, these findings suggest that augmentation with HA granules is safer than conventional augmentation using cement or expandable screws.

The present study has some limitations. First, biomechanical tests cannot completely simulate the physiological condition of the lumbar spine in patients after surgery using PPS fixation. In the clinical setting, multiple pedicle screws are inserted at various angles connected with rods, which is likely to affect the stability of individual screws. In addition, various factors, such as the diameter, length and trajectory of the screw as well as the design of the screw-thread, tapping size and bone quality and morphology of each patient, are likely to affect the outcome, so the present findings should be interpreted carefully. Second, the current study used synthetic bone for mechanical tests, so the results might be different in a study using cadaveric models. However, many previous studies have used the same polyurethane foam model as was used in this study to perform mechanical tests of pedicle screw fixation.[43, 48, 54] Those studies provided valuable data as good as those obtained in studies using cadaveric models. Indeed, the ASTM protocol for biomechanical studies recommends the use of polyurethane foam model because synthetic bone with uniformity and consistent properties is more suitable than cadaveric bone for comparative mechanical testing of screw fixation.[47] Third, the results of mechanical testing using the synthetic bone model cannot directly reflect biological effect of HA granules *in vivo*. However, the insertion of HA granules into the screw hole actually has a mechanical effect to increase the bone-metal interface friction force and enhances the initial stability of screw fixation.[21, 23] Therefore, the findings of the present study using the polyurethane foam should be resulted from the mechanical effect of HA granules to improve the initial strength of screw fixation. Although these limitations must be considered when interpreting the results, the current study produced valuable data supporting the mechanical advantages of augmentation of PPS fixation with HA granules. The present study used the polyurethane foam having the density of 0.08 g/cm^3^ in mechanical testing in order to confirm the effectiveness of the augmentation using HA granules in severe osteoporotic bone. The results might be different in a study using polyurethane foams with either higher or lower density. Further studies may clarify the difference in the effectiveness of augmentation using HA granules among various bone qualities.

## Conclusion

The present study demonstrated that augmentation with HA granules significantly enhanced the pullout strength and stiffness of PPS fixation using an osteoporotic bone model. In addition, the insertion torque of screws with augmentation was significantly greater than that without augmentation. Furthermore, the resistance to cyclic loading was significantly stronger in screws with augmentation. These findings suggested that augmentation using HA granules is effective for increasing the strength and stiffness of PPS fixation in osteoporotic bone. The present study reported that novel augmentation using HA granules may be a useful technique for PPS fixation.
